# Approaches to the rationalization of surgical instrument trays: scoping review and research agenda

**DOI:** 10.1186/s12913-021-06142-8

**Published:** 2021-02-20

**Authors:** Bruno Miranda dos Santos, Flavio Sanson Fogliatto, Carolina Melecardi Zani, Fernanda Araujo Pimentel Peres

**Affiliations:** Department of Industrial Engineering, Federal University of Rio Grande do Sul, Av. Osvaldo Aranha, 99, 5° andar, Porto Alegre, 90035-190 Brazil

**Keywords:** Surgical tray rationalization, Surgical instruments, Surgical trays, Processes improvement, Scoping review

## Abstract

**Background:**

Surgical Tray Rationalization (STR) consists of a systematic reduction in the number of surgical instruments to perform specific procedures without compromising patient safety while reducing losses in the sterilization and assembly of trays. STR is one example of initiatives to improve process performance that have been widely reported in industrial settings but only recently have gained popularity in healthcare organizations.

**Methods:**

We conduct a scoping review of the literature to identify and map available evidence on surgical tray management. Five methodological stages are implemented and reported; they are: identifying research questions, identifying relevant studies, study selection, charting the data, and collating, summarizing and reporting the results.

**Results:**

We reviewed forty-eight articles on STR, which were grouped according to their main proposed approaches: expert analysis, lean practices, and mathematical programming. We identify the most frequently used techniques within each approach and point to their potential contributions to operational and economic dimensions of STR. We also consolidate our findings, proposing a roadmap to STR with four generic steps (prepare, rationalize, implement, and consolidate) and recommended associated techniques.

**Conclusions:**

To the best of our knowledge, ours is the first study that reviews and systematizes the existing literature on the subject of STR. Our study closes with the proposition of future research directions, which are presented as nine research questions associated with the four generic steps proposed in the STR roadmap.

## Background

Two main concerns seem to permeate the healthcare industry worldwide [1] reduction of public investments and rise in care demand. According to OCDE [2], while government spending in health remained constant at average 8.8% of the GDP from 2013 to 2018, demand for healthcare services is continuously increasing, resulting in overcrowded systems that potentially expose patients to risks and compromise the quality of care. Recent evidence of healthcare systems operating at maximum capacity was brought by the COVID-19 outbreak, a public health emergency of global scale [3]. In such contexts, managerial efforts to optimize healthcare processes become particularly recommendable to increase efficiency and promote system-level resilience. The analysis of healthcare supply chains reveals several opportunities for improvements in processes often deemed secondary. One such process is the sterilization and assembly of surgical trays, with improvement initiatives usually targeted at the rationalization or optimization of tray contents [4].

Surgical Trays (ST) are containers that hold surgical instruments [5]. Each ST contains the instruments needed to perform a surgical procedure or family of procedures. When the ST configuration is properly designed, it offers a minimum set of required instruments to perform the highest number of procedures within a specialty [6]. ST management is centered on four main questions: (i) which instruments should be placed on the STs; (ii) in what quantities; (iii) which STs are used in which surgical procedures; and (iv) how many STs of each type should be held in inventory. While (i) and (ii) are affected by surgeons’ preferences, (iii) and (iv) depend on the frequency and scheduling of procedures in the surgical center [1].

STs are used in Operating Rooms (OR) along with other supplies such as clothing and individual instruments. All materials supplied to ORs must be sterile and may be classified as disposable (dispensed after a single use) or reusable items (which must be sterilized before reuse). The use of disposable items may be justified by convenience and safety, but they usually come at a higher cost, favoring the adoption of reusable items [7, 8]. ORs are cost-intensive units [9] to which redundant or unused materials are constantly supplied. Empirical research has shown that improving surgical instruments’ management offers an opportunity to significantly reduce OR costs [1, 10, 11].

Keeping the balance between the capacity of resources and a fluctuating and uncertain demand is a challenge for managers of healthcare systems [12, 13]. Materials from a central processing sterilization plant, in particular, are demanded by several sectors within the hospital that may compete for the same items [14]. The problem grows in complexity if lack of process standardization regarding surgical tray assembly and poor material distribution logistics are considered [15].

Studies suggest that elimination of unnecessary or redundant instruments in STs may promote time savings [16], less operational effort [17], and smaller cost in ORs [18], without negative effects to patients [19–21]. Rationalization of surgical instruments may also significantly reduce sterilization time [22], ergonomic risks [1], and unnecessary purchases of instruments [21].

Despite the number of studies exploring reduction in the number of surgical instruments in STs, the topic is relatively new in the literature, with many open research opportunities for more robust solutions. The first step towards that is a comprehensive analysis of existing approaches. That is the main motivation for this article: to investigate approaches and techniques available in the literature for reducing instruments in STs. For that, we present the results of a scoping review aiming at identifying and mapping available evidence on the topic of ST management [23]. Scoping reviews are suggested as a complement to systematic reviews when the literature on the subject of interest is scarce and complex [23].

To the best of our knowledge, this is the first article that comprehensively covers state of the art on Surgical Tray Rationalization (STR), adding to the work of Dekonenko et al. (2020), who conducted a literature review covering five studies describing techniques for STR, limited to the pediatric surgical specialty. In addition to the description of approaches and techniques for rationalizing STs, we also included relevant data on results obtained in the cases reported, such as the percentage of reduction in instruments and gains in operational and economic performance. A table summarizing the most frequently used STR techniques and their impact on operational and economic healthcare dimensions is also proposed. The study also provides a flowchart of STR stages, indicating analytical techniques more suitable at each stage. The paper closes with the proposition of a future research agenda.

## Methods

We follow the methodological stages proposed by Arksey and O’Malley [23] for scoping studies; namely: (1) identifying research questions; (2) identifying relevant studies; (3) study selection; (4) charting the data; and (5) collating, summarizing and reporting the results. A scoping literature review maps adjacent concepts related to an area of interest and main sources of information available [23, 24], being grounded on wide questions to investigate emerging evidence that could not be compiled through a systematic literature review [25, 26].

### Identifying research questions

The following adjacent concepts related to STR motivated the research questions and guided the structuring of results and their discussion: managerial approaches and predominant techniques for instrument reduction, indicators of instrument reduction, and reported impacts on performance dimensions (both operational and economic). We organized the scoping study around four research questions:

**RQ1.** What is the existing literature on STR in healthcare organizations?

**RQ2.** What are the main approaches and techniques reported for STR?

**RQ3.** What are the areas impacted by STR, considering operational and economic performance?

**RQ4.** What are the research gaps in the STR literature that could be organized in a future research agenda?

Research questions were formulated following the guidelines proposed by Mays et al. [27] and answered using a structured system based on the PRISMA (Preferred Reporting Items for Systematic Reviews and Meta-Analysis) model [28] and on scoping review principles [23].

### Identifying relevant studies

Four databases were searched to identify works available on ST management: Scopus, PubMed, Web of Science (WOS), and ScienceDirect (SD). They are identified by Augusto and Tortorella [29] and Tortorella et al. [30] as predominant in review studies in the healthcare area. Keywords were selected from ten articles reported in Fogliatto et al. [6], whose study is aligned with our research questions. We carried a two-step search in the databases. A primary search looked for articles with the words “surgical tray”, “surgery tray” and “instrument tray” in titles, abstracts and/or keywords. A secondary search included the words “instrument* reduc*”, “tray* config*”, “tray* optim*”, “surger* instrument*”, and “tray* redundanc*”. The last search was carried out in October 2020. A consensus-based Boolean search strategy was created by the authors to consider possible word synonyms in the secondary search in Scopus, WOS, and Pubmed databases. The SD database does not enable this search strategy, and word combinations were inserted manually.

### Study selection

**Fig. 1:**
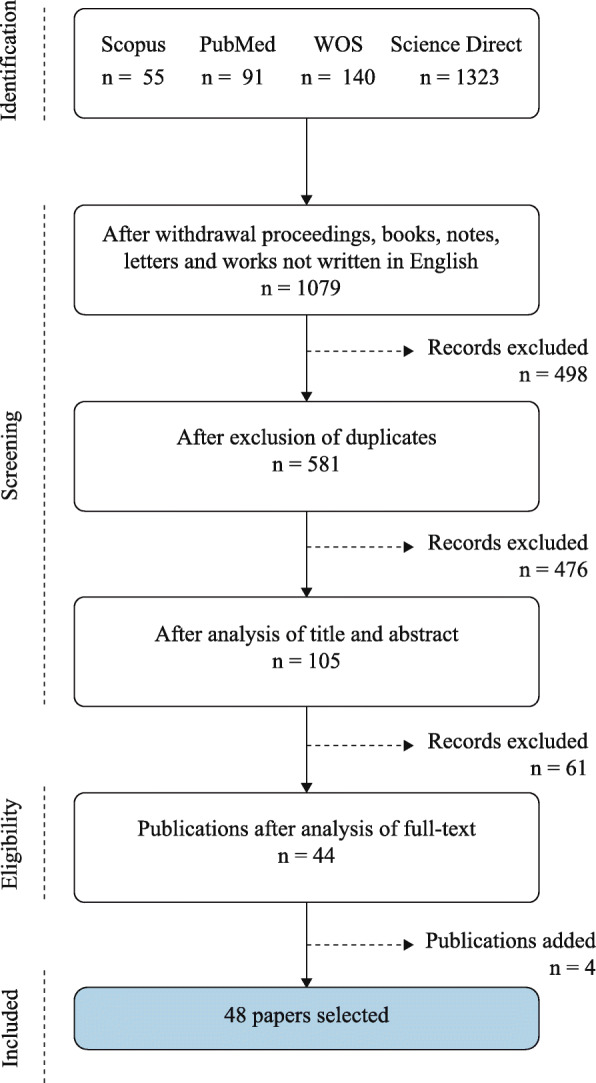
Results from PRISMA stages

The selection process was carried out concurrently by two of the paper’s authors, as recommended by Munzer et al. [31]. They followed the PRISMA stages [28] of identification, screening, eligibility, and inclusion, leading to the results in Fig. 1. In the screening stage, different inclusion/exclusion criteria were formulated post hoc, as the researchers’ familiarity with the literature increased. Only articles published in English and peer-reviewed were considered. Inclusion criteria comprised observational or interventional studies reporting quantitative, qualitative, or mixed approaches. Exclusion criteria were articles with titles and abstracts not aligned with our research questions, and articles focusing on cleaning instruments, contamination reduction, cleaning guidelines, sector layout, professional education, and types of washing. A full-text analysis was carried out in the eligibility stage, yielding 44 articles that were considered fully aligned with the proposed research questions. Browsing references from those articles, 4 additional works were selected to compose the final corpus of publications considered in this scoping study, totaling 48 articles.

### Charting the data

The objective here is to map and interpret key data from selected articles and visualize existing relationships that may be considered in the next stage [32]. Information extraction used a descriptive-analytical method [23, 33] to provide a holistic view of the works selected. Seven types of information were searched in the corpus of articles to characterize tray management practices used in hospitals, as described in Table 1.

**Table 1:** Types of information searched in the selected corpus of articles

Type	Extracted contents
Bibliometric information	Authors; journal; JCR Impact factor; year of publication; country where the study was developed
Surgical specialty	Specialties considered in the study
Actors	Professional profiles of those involved in improvement initiatives
Approach	Type of approach used in improvement initiatives (e.g., based on lean principles, linear programming and heuristics or expert analysis)
Rationalization/ optimization techniques	Techniques used within the adopted approach and results obtained
Barriers and challenges	Main barriers for implementation of techniques and challenges that emerged
Future research	Main knowledge gaps

### Collating, summarizing, and reporting the results

In this stage, results and extracted information are presented in a thematic framework that answers the research questions. Here, the terms ‘reduction of surgical instruments’ and ‘reduction of surgical trays’ were unified and denoted as “Surgical Tray Rationalization” (STR). As prescribed by Levac et al. [32], two complementary analyses were carried out to increase the consistency of results. The first comprises the elaboration of a descriptive numerical summary, followed by thematic analysis. Numerical results provide information on the corpus’ main characteristics (e.g., total number of studies, year of publication, analyzed specialties), contributing to address RQ1 and providing insights for RQ2. Thematic analysis provides an understanding of approaches and techniques used for STR, addressing RQ2. The identification of impacted areas by each approach and technique, at both operational and economic levels, addresses RQ3 and provides insights for RQ4. In the second analysis, we propose a structure for implementing STR techniques based on targeted results (detailed in sections 4 and 5) that organizes recommendations dispersed in the existing literature. Implications of our findings were discussed, ensuring the legitimacy of the study’s scope methodology for theory and practice and allowing the proposition of a future research agenda with research questions associated with the STR steps proposed in section 5, and addressing RQ4.

## Results

**Fig. 2:**
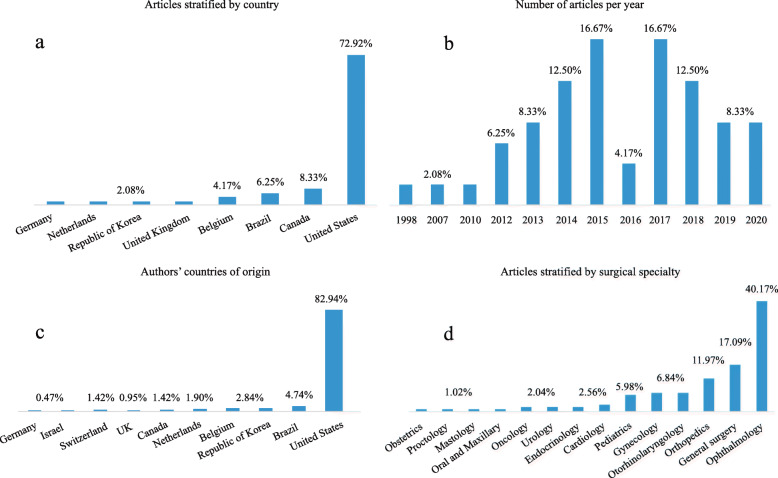
Quantitative summary of the selected corpus

Figure 2 shows a quantitative summary of the selected corpus, answering RQ1. The 48 selected articles were written by 211 authors. Only 22 (10%) of them participated in two or more studies, never in the same journal. Dispersion of authors and lack of a specific vehicle that concentrates studies is common in research topics about which knowledge is still incipient. Multinational research teams authored only three studies: Belgium and the United Kingdom in Cardoen et al. [34], Germany, Switzerland, and the Netherlands in Tibesku et al. [35], the USA and Israel in Yoon et al. [22]. A high concentration of authors (82.94%, *n* = 175, Fig. 2c) and articles (72.92%, *n* = 35, Fig. 2a) on STR come from the USA, which is not surprising given that it is the country that most spends in healthcare [36], justifying the interest in waste reduction initiatives. Most authors are associated with the Healthcare knowledge area (84.83%, *n* = 179), followed by Engineering (8.53%, *n* = 18). Articles authored by healthcare researchers mostly neglect traditional concerns from the Operations Management field, such as improvement standardization actions and control and monitoring of improvements already implemented.

Journals targeted by authors also reflect their main expertise area: 94.29% of the articles were published in healthcare journals. Authors tend to cooperate with others from the same area producing STR propositions that are often poor as managerial improvement reports. STR studies published in Operations Management journals (e.g. *IIE Transactions on Healthcare Systems Engineering*, *European Journal of Operational Research* and *Production Planning & Control*) usually team authors with different backgrounds (e.g. Engineering, Management, Design) and are methodologically better structured. The Journal of Arthroplasty published the largest number of articles in our corpus (*n* = 4), although focusing on the use of a patient-customized ST to perform a single procedure (Total Knee Arthroplasty) targeting at trays with fewer instruments (e.g. ) [37] that would lead to reduced operating times (e.g. ) [35].

The corpus of articles on STR covered 14 surgical specialties and 116 procedures. Most procedures analyzed belonged to the Ophthalmology specialty (40.17%, Fig. 2d), which is volume intensive (i.e., short procedures) and characterized by STs with a large number of instruments. Studies that did not mention the specialty analyzed were classified as General Surgery, which appears as the second most investigated (17.09%, *n* = 20), followed by Orthopedics (11.97%, *n* = 14).

Figure 2b shows the evolution in the number of articles on STR per year, with around 50% published in the past 5 years. The first study reporting the benefits of STR was published in 1998 in the Obstetrics and Gynecology Journal (JCR = 4.965). The second study, following a gap of more than 10 years, was published in 2007 in the Journal of the American Medical Association (JCR = 10.668). From 2012 on, the subject of STR became increasingly present in the literature.

We now present a thematic analysis of our corpus. We classified STR studies according to their main approach, as follows (Table 2): (i) expert analysis (EA), (ii) lean practices (LP), and (iii) mathematical programming (MP). EA approaches are predominant, accounting for 70.83% (*n* = 34) of the articles, all of them published in healthcare journals. Most EA approaches were proposed by authors from the healthcare field, with a broad knowledge of the surgical specialties addressed. LP approaches are the second more frequent (18.75%, *n* = 9), with articles mostly published in healthcare journals [*n* = 8; the exception is Fogliatto et al. [6] published in *Production Planning & Control*]. MP approaches are the least frequent (10.41%, *n* = 5), with authors presenting greater background diversity (Engineering, Computing, and Healthcare). Most studies targeted a single surgical specialty, and reported rationalizations derived from surgical team meetings and discussions using simple techniques such as consensus groups and checklists, often supported by historical data analysis.

**Table 2:** Classification of STR studies according to main approach

*Journal title*	*Number of articles*	*Clusters identified*		
		Expert Analysis	Lean Practices	Mathematical programming
The Journal of Arthroplasty	4	[37, 40, 41, 45]	–	–
Journal of Pediatric Surgery	3	[15, 67, 68]	–	–
Journal of Surgical Research	3	[4, 46, 69]	–	–
Otolaryngol - Head and Neck Surgery	2	[50, 55]	–	–
Journal for Healthcare Quality	2	–	[16, 57]	–
IIE Transactions on Healthcare Systems Eng	2	–	–	[1, 61]
Journal of Pediatric Urology	2	[18]	[60]	–
Archives of Orthopaedic and Trauma Surgery	2	[35, 42]	–	–
American Journal of Obstetrics and Gynecology	2	[48, 66]	–	–
International Journal of Retina and Vitreous	1	[49]	–	–
Revista Brasileira de Enfermagem	1	–	–	[63]
Obstetrics and Gynecoloy	1	[64]	–	–
JAMA Surgery	1	–	[56]	–
AORN Journal	1	[65]	–	–
International Journal of Gynecology and Obstetrics	1	[19]	–	–
Orthopedics	1	[44]	–	–
Journal of Otolaryngology - Head & Neck Surgery	1	[54]	–	–
The Spine Journal	1	–	[20]	–
Surgery	1	[52]	–	–
The Knee Journal	1	[43]	–	–
Journal of the American College of Surgeons	1	[38]	–	–
International Journal of Production Research	1	–	–	[34]
Diseases of the Colon and Rectum	1	[70]	–	–
Journal of Hospital Administration	1	[10]	–	–
Laryngoscope	1	–	[58]	–
CMAJ Open	1	[47]	–	–
Journal of Cardiology & Cardiovascular Therapy	1	[53]	–	–
Annals of Thoracic Surgery	1	–	[59]	–
Journal of Gynecologic Surgery	1	[21]	–	–
European Journal of Operational Research	1	–	–	[62]
Plastic and Reconstructive Surgery	1	[17]	–	–
American Journal of Medical Quality	1	–	[22]	–
Medicine	1	[39]	–	–
Production Planning & Control	1	–	[6]	–
Journal of Minimally Invasive Gynecology	1	[51]	–	–

[1]Ahmad et al. (2019) [4]; Malone et al. (2019) [6]; Fogliatto et al. (2020) [10]; Mhlaba et al. (2015) [15]; Avansino et al. (2013) [16]; Fogliatto et al. (2018) [17]; Humphries et al. (2018) [18]; Nast and Swords (2019) [19]; Greenberg et al. (2012) [20]; Lunardini et al. (2014) [21]; Harvey et al. (2017a) [22]; Yoon et al. (2018) [34]; Cardoen et al. (2015) [35]; Tibesku et al. (2013) [37]; Siegel et al. (2015) [38]; Stockert and Langerman (2014) [39]; Capra et al. (2019) [40]; Dehann et al. (2014) [41]; Hamilton et al. (2013) [42]; Kwon et al. (2017) [43]; Renson et al. (2014) [44]; Hsu et al. (2012) [45]; McLawhorn et al. (2015) [46]; Farrely et al. (2017) [47]; John-Baptiste et al. (2016) [48]; Van Meter and Adam (2016) [49]; Grodsky et al. (2020) [50]; Penn et al. (2012) [51]; Harvey et al. (2017b) [52]; Morris et al. (2014) [53]; Barua and O’Regan (2017) [54]; Chin et al. (2014) [55]; Crosby et al. (2020) [56]; Bush (2007) [57]; Farrokhi et al. (2013) [58]; Wannemuehler et al. (2015) [59]; Cichos et al. (2017) [60]; Koyle et al. (2018) [61]; Dobson et al. (2015) [62]; Dollevoet et al. (2018) [63]; Schneider et al. (2020) [64]; Bachmann et al. (1998) [65]; Ngu (2010) [66]; Byrnes et al. (2017) [67]; Robinson et al. (2018) [68]; Skarda et al. (2015) [69]; Dyas et al. (2018) [70]; Guzman et al. (2015)

We address RQ2 through Table 3, which summarizes STR approaches and the percentage of instrument reduction attained. Most studies (*n* = 20) report reductions greater than 50%, 9 report reductions between 26 and 50%, and in 7, reductions below 25%. No study failed at obtaining improvements in the tray rationalization process, although some did not report quantitative indicators. Classification of techniques was primarily based on authors’ declarations. For example, techniques usually associated with Lean Production were only classified under the LP approach if the study was framed in a lean context. Otherwise, they were classified as EA. In the MP category, studies presented some mathematical formulation of the STR problem.

**Table 3:** Techniques used in each STR approach and percentage of instrument reduction reported

Approach	Author	Technique	Reduction	Approach	Author	Technique	Reduction
AE	[4]	CL, OBS, STD	48.00%	AE	[53]	CL, OBS	39.50%
AE	[10]	FG, OBS, CA, CT, CP	61.00%	AE	[54]	CL, FG	57.00%
AE	[15]	STD, PC, CL, OBS, FG	NR	AE	[55]	CL	51.92%
AE	[17]	CT	NR	AE	[64]	FG, SM, SIT	33.33%
AE	[18]	CT, PDSA, FG, RCA, KDD	38.60%	AE	[65]	PC, FG, CL	63.74%
AE	[19]	STD	NR	AE	[66]	CL, STD	NR
AE	[21]	CL, FG	19.10%	AE	[67]	STD, PC, OBS	NR
AE	[35]*	ABC, PSI, ITV	66.67%	AE	[68]	STD, PC, FG	NR
AE	[36]	FG, CL	30.00%	AE	[69]	CL, STD	63.27%
AE	[37]	PSI, OBS	50.00%	AE	[70]	CT	NR
AE	[38]	OBS, ITV, CL, CA	13.50%	PL	[6]	FG, CAN, SOP, CL	10.78%
AE	[39]	FG, OBS, PSI	62.11%	PL	[16]	CAN, KAI	11.00%
AE	[40]*	CL, PSI	66.67%	PL	[20]	CL, OBS, STD, KAI	41.45%
AE	[41]*	PSI	62.50%	PL	[22]	FG, CL, SXS	9.86%
AE	[42]*	PSI	54.55%	PL	[26]	STD, PC, CL, OBS, FG	76.87%
AE	[43]	PSI	54.55%	PL	[56]	OSW, SIT, KAI	NR
AE	[44]*	TDI	57.14%	PL	[57]	VSM, L5S, STD, OBS, KAI, FG, CL	70.56%
AE	[45]*	TDI, DT	42.86%	PL	[58]	SXS, FG, VSM, KAI, OSW, CL, STD	53.85%
AE	[46]	OBS, STD, FG, BRT, CL	62.30%	PL	[59]	STD, FG, CL	58.74%
AE	[47]	CT	58.00%	MP	[1]	MILP, HEU, PC	NR
AE	[48]	OBS, CL, CA	46.67%	MP	[12]	PLM, HEU	NR
AE	[49]	CL, STD	89.00%	MP	[34]	CL, HEU, NIP, CP	NR
AE	[51]	PC, CL, FG	NR	MP	[61]	PLM, HEU	61.59%
AE	[52]	NG, PF, RCA, FG, SOP, CL	20.00%	MP	[63]	BRT, OBS, FG, CAN, LP	13.10%

Approaches: *AE* Expert Analysis, *LP* Lean Practices, *MP* Mathematical programming. Techniques: *ABC* ABC costing, *BRT* Brainstorming, *CA* Chrono-analysis, *CAN* Closest neighbor algorithm, *CL* Check-list, *CP* Custom pack, *CT* Cost tray, *DT* Decision tree, *FG* Focus groups, *HEU* Heuristic, *ITV* Interview, *KAI* Kaizen, *KDD* Key-driver diagram, *L5S* Lean 5S, *LP* Linear programming, *MILP* Mixed integer linear programming, *NG* Nominal group, *NIP* Nonlinear integer programming, *OBS* Observation, *OSW* Ohno’s seven wastes, *PC* Preference cards, *PDSA* Plan, Do, Study, Act, *PF* Process flowcharts, *PLM* Modified integer linear programming, *PSI* Patient-specific instrumentation, *RCA* Root cause analysis, *SIT* Shadowed instrument tray, *SM* Surgical manual, *SOP* Standard operational procedure, *STD* Tray standardization, *SXS* Six sigma, *TDI* Template-directed instrumentation, *VSM* Value Stream mapping, *NR* Not reported; (*) Indicates reduction in the number of STs.

Tibesku et al. [35] and Stockert and Langerman [62] were the most cited articles from the EA category (based on Scopus citations). Tibesku et al. [35] analyzed the cost benefits of implementing Patient Specific Instrumentation (PSI) to perform Total Knee Arthroscopy. To define PSI, authors used interviews (ITV) with surgeons; cost benefits were assessed through ABC costing. PSI reduced the number of STs by 66.67%, leading to smaller sterilization, maintenance, and storage costs. Other articles that used PSI alone or in combination with other techniques were mostly focused on cost reduction, although reporting secondary benefits such as reductions in ST weight [69], OR setup time [38], the total time to perform the operation [39], and OR infection rates [37], and improvement in mechanical alignment [50, 61]. Hsu et al. [58] and McLawhorn et al. [40] also reported significant cost reductions in Total Knee Arthroscopy derived from using Template-directed instrumentation (TDI) and a combination of TDI and decision trees (DT). In opposition to PSI, which uses disposable items, TDI uses conventional reusable instruments combined with PSI instrument reduction principles.

Stockert and Langerman [62] reported an average 13.50% reduction in instruments used in four surgical specialties by combining ITV, observation (OBS), checklists (CL), and chrono-analysis (CA) methods to identify redundant instruments in STs. The authors estimated the processing cost per instrument, which was used in several other STR studies [4, 10, 43, 51, 53, 65].

The most widely used technique in STR studies based on EA was the CL, followed by focus groups (FG), OBS, and standardization (STD). FG were an important means to motivate multidisciplinary groups to analyze ST rationalization through observation of actual instrument usage, reviewing preference cards, creating educational programs to minimize waste, and assessing team motivation after implementation [15, 21, 45, 60, 70]. The indicator utilization rate was used by several authors (e.g.) [46, 59, 66]. Other indicators included sterilization cost, ST weight reduction, and OR setup time reduction.

Among studies classified in the LP category, the most cited is Bush [56], followed by Farrokhi et al. [47] and Lunardini et al. [20]. Bush [56] used lean to reduce losses in a large medical center, following Ohno’s seven waste categories (OSW). To reduce errors due to wrong or missing instruments in the assembly of STs, a shadowed instrument tray (SIT) was adopted. Improvement cycles were proposed through kaizen groups (KAI). OSW combined with value stream mapping (VSM) was also the analytical framework adopted by Wannemuehler et al. [64]. They mapped the complete cycle of ST’s utilization, identifying activities that did not comply with users’ requirements and proposing improvements through KAI, to obtain a 53.85% reduction in instruments in the STs analyzed.

Farrokhi et al. [47] combined OBS, CL, VSM, KAI, Lean 5S (L5S) and STD to analyze back surgery trays. L5S implementation was guided by an interdisciplinary FG with the objective of identifying the usage rate and availability of instruments, as well as those obsolete. New STs were proposed and monitored in the ORs. Two outcomes were reported: a reduction of 70% in the number of instruments supplied to the ORs and shorter surgery times. Lunardini et al. [20] combined CL, OBS, STD, and KAI to obtain an average instrument reduction of 41.45% in STs supplied to the orthopedics specialty. Fogliatto et al. [6, 16] analyzed STs from a larger number of surgical specialties (e.g., ophthalmology, urology, and pediatrics), which were grouped using the closest neighbor algorithm (CAN) and rationalized through a standard operational procedure (SOP) performed within KAI groups. They reported a reduction of 10.78% in instruments overall specialties analyzed. Farrokhi et al. [47], Wannemuehler et al. [64], and Fogliatto et al. [16] analyzed differences in indicators (e.g., waiting times, ST assembly times) before and after rationalization using statistical tests.

The most widely used technique in studies classified in the LP category is CL, followed by FG, KAI, and STD. Instrument utilization rate is an indicator frequently reported [22, 49, 67] in the context of VSM, when instruments are categorized as needed (i.e., value-adding) or not needed [47, 64]. Another frequently reported indicator is the satisfaction level of those involved in the rationalization process, higher in studies involving multidisciplinary groups and covering several surgical specialties. For example, Koyle et al. [49] and Yoon et al. [22] reported high satisfaction levels with no instruments added to trays after revision; that was not the case in Farrely et al. [43], in which few specialties enrolled in the rationalization effort. In general, reaching consensus among surgical teams increased the observed satisfaction level after ST revision [64].

Among studies classified in the MP category, Dobson et al. [48] is the most cited, followed by Cardoen et al. [34]. Dobson et al. [48] used Modified Integer Linear Programming (PLM) and Heuristics (HEU) to find the composition of STs that minimized costs in the operation of a surgical center, satisfying surgeons’ instrument and scheduling preferences. They reported a reduction of 61.59% in instruments when customizing STs to a particular surgical schedule (although greatly increasing ST assembly complexity). Dollevoet et al. [68] modeled the same problem as [48], testing the performance of several heuristics with respect to computational time and quality of the solution provided for short and long planning horizons. Results recommended the use of heuristics for short horizons and of PLM, otherwise. Both studies confirmed better cost reduction performance when a small number of surgical specialties is scheduled to operate on the same day at the surgical center due to sharing of instruments.

Cardoen et al. [34] optimized the configuration and assignment of STs to surgical procedures using Nonlinear Integer Programming (NIP), CL, and HEU. They analyzed (i) the feasibility of using custom packs (CP) of instruments, which may reduce the overall number of items used to perform surgeries but may be unpractical in terms of assembly, and (ii) the increase in sharing of STs among surgical procedures resulting from adding redundant instruments to trays. They addressed the analysis in (i) using HEU. Ahmadi et al. [1] used Mixed Integer Linear Programming (MILP), preference cards (PC), and HEU to configure STs considering ergonomic risks. In opposition to other authors, they reviewed lists of instruments in PCs to identify those obsolete for removal.

Schneider et al. [54] combined techniques belonging to the EA and LP categories (e.g., FG, OBS, and BRT) with linear programming (LP) and CAN to review instruments used in 20 ophthalmology procedures and identify infeasibilities in their scheduling in the surgical center. Results included the reduction in the total number of instruments in STs and an increase in the number of procedures performed, although numerical figures were not given.

The most frequently used technique in studies classified in the MP category is HEU; all other MP techniques were used at least once. Instrument utilization rate along with the availability of personnel, OR, individual instruments, and STs were information used in all MP studies [1, 34, 48, 54, 68]. When HEU was combined with techniques from other approaches (e.g., EA and LP), the likelihood of solving the ST configuration problem increased.

**Fig. 3:**
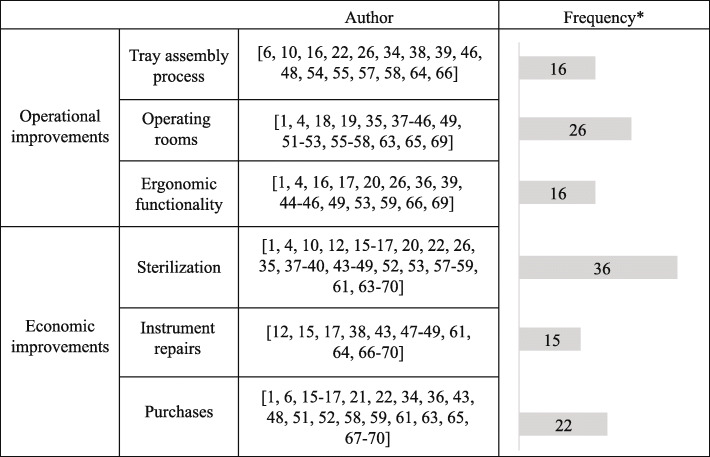
Operational and economic improvements reported in STR studies. (*) frequency indicates the number of publications reporting each operational and economic improvement

In Fig. 3, we list operational and economic dimensions impacted by ST instrument reduction, as reported by authors from our corpus. Three operational improvements are listed: (i) tray assembly process; (ii) operating rooms (ORs); and (iii) ergonomic functionality associated with the person handling the tray (e.g., size, weight). Three economic improvements are listed: cost reduction in (i) sterilization; (ii) instrument repairs; and (iii) purchases. No article reported all six types of improvements. Within the operational dimension, improvements in ORs were the most frequently mentioned (*n* = 26). Within the economic dimension, sterilization of instruments (*n* = 36) and cost reduction with purchases (*n* = 22) were the most frequently mentioned. Results in Fig. 3 provide the answer to RQ3.

Regarding the operational dimension, improvements in (i) are usually associated with faster processing of trays, from cleaning to assembly, due to the smaller number of instruments. That promotes process agility and reduces assembly errors [69]. Although impacting the economic dimension, we classified such improvement as operational since most authors do not report savings associated with the assembly process (an exception is Fogliatto et al. [6]. Improvements in (ii) are related to actions before (open and check STs), and after (replace instruments in STs and check again) the procedure takes place in the OR. There is an economic aspect to this improvement as well; however, most authors associate it with a reduction in time to set up [4, 46] and count instruments [62], and errors handling instruments to surgeons [56]. Improvements in (iii) are ergonomic. It is known that weight is one the main risk factors associated with the handling of STs [1].

Regarding the economic dimension, improvements in (i) are related to the sterilization of STs and instruments. Rationalized STs are smaller, increasing the number of trays processed in the same autoclave batch and reducing unitary costs [51]. Improvements in (ii) are related to costs with maintenance due to improper placement of instruments on trays and high frequency of sterilization cycles, causing excessive wear [55] and depreciation due to use [66]. Improvements in (iii) are related to purchasing new or replacement instruments. Manufacturers usually recommend replacement or maintenance of instruments after a given number of sterilization cycles [70]; therefore, a reduction in the number of cycles may lead to significant savings [57, 70].

## Discussion

**Fig. 4:**
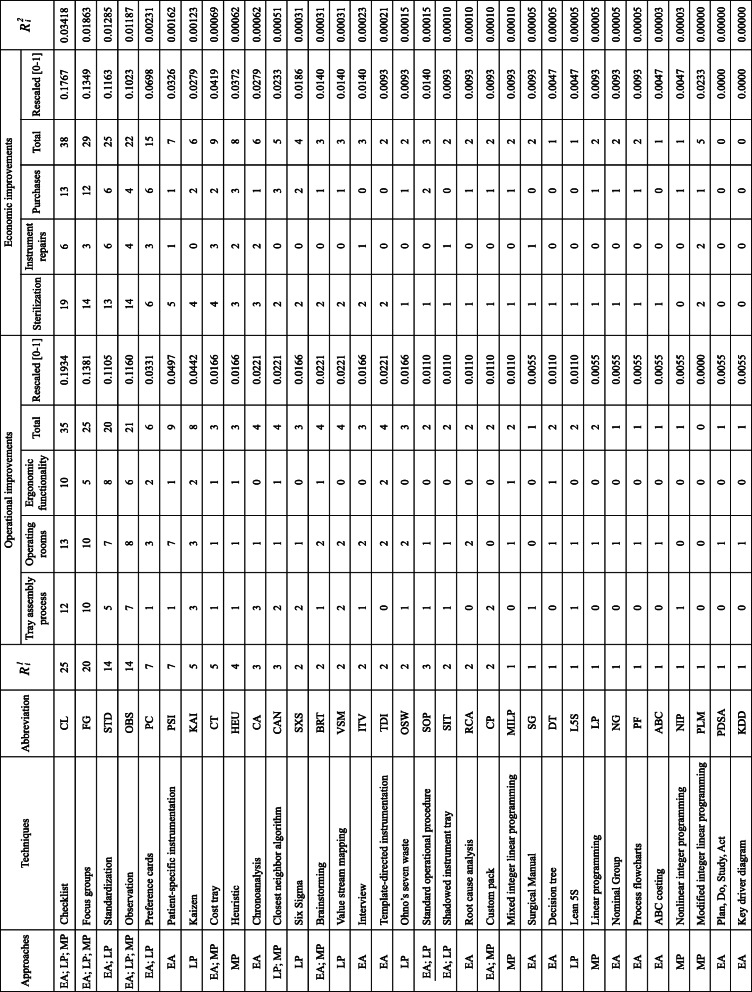
Improvements and importance scores associated with STR techniques. Columns: 1–3: approaches, techniques, and abbreviations; 4: ${R}_i^1$: number of works that reported the use of technique; *i;* 5–7: number of works that reported impacts of each technique on operational improvements; 8: sum of entries in columns 5 to 7; 9: normalizes the values in column 8 onto a [0,1]-scale 10–14: repeat the same structure as columns 5 to 9 but focusing on economic improvements 15: ${R}_i^2$: score of classification of techniques *i* in relation to operational and economic improvements

Figure 4 summarizes our findings on approaches and techniques used in STR. It is important to remark that most articles report the combined use of more than one technique. We now present details on the top three most frequently used techniques.

CL is the technique most frequently used in the corpus of papers (*n* = 25); it is also the one with the largest ${R}_i^2$ value. A CL is a list of instruments needed to properly execute a surgery. Stockert and Langerman [62] is the most cited paper among the ones that use CL. It uses the technique in two moments: before surgery, to revise ST contents, and after surgery, to identify instruments not used. In the study, the use of CL is combined with ITV, OBS, and CA. They reported improvements in instruments’ maintenance and OR setup, among others.

FG, the second most frequently used technique (*n* = 20) and the second-largest ${R}_i^2$ value, is a form of interview based on communication and interaction with groups of individuals, preferably with different backgrounds. The objective is to gather detailed information on a specific subject. Among studies that report the use of FG, Farrokhi et al. [47] is the most cited. In the study, FGs are carried out with a kaizen group formed by nurses, nurse technicians, surgeons, and sterilization technicians to evaluate ST rationalization, which was implemented using lean principles (e.g., VSM, L5S, and STD). They reported improvements in tray assembly and time to complete surgical procedures, among others.

STD and OBS are the third most frequently used techniques in STR studies, each with 14 works, although benefits from using STD are more numerous than those of using OBS, as given by their ${R}_i^2$ values. STD sets a common ground on instruments needed to perform a given surgical procedure, enabling the assembly of a rationalized ST that will be used by most surgeons. OBS is a data collection technique usually carried out by nurse technicians aimed at gathering information on instrument utilization. In several cases, OBS is used jointly with CL directly in the ORs [43, 49]. Farrokhi et al. [47] and Stockert e Langerman [62] were the most cited articles using STD and OBS, respectively. Farrokhi et al. [47] used OBS in spine surgeries to identify the most frequently used instruments, followed by STD, to create a basic surgery tray for the procedures. In Stockert and Langerman [62], OBS was used in the ORs to identify frequently used instruments and in the sterilization center, to determine the time needed to clean instruments and assemble STs. It is noteworthy that STD is used differently by authors following LP and EA approaches. In LP, STD is the basis for continuous improvement, being sought in all steps of STR [47, 49]; in EA, STD corresponds to the last step of an STR analysis [41, 52].

The FG and STD techniques most commonly used are aligned with findings in Dekonenko et al. (2020); however, their study focused on a single surgical specialty (pediatrics), with results that are difficult to generalize. As reported in our review, the five studies presented in Dekonenko et al. (2020) promoted significant cost savings, ergonomic advantages, and greater agility in the surgical process. Our work, however, considers a larger number of studies and specialties, comprehensively detailing each technique and its benefits. We also analyzed each technique from a two-dimensional perspective (operational and economic), classifying their derived improvements, as shown in Fig. 4.

### Proposed steps for STR studies

**Fig. 5:**
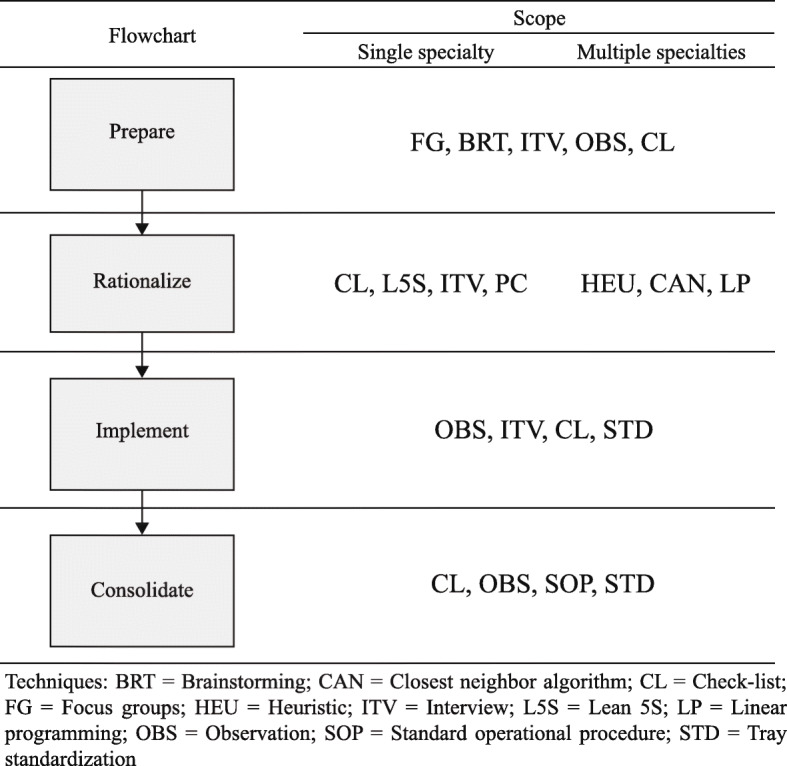
Flowchart of the tray rationalization process steps

Based on the analysis of STR approaches in our corpus, we propose a four-step general flowchart (Fig. 5) for STR studies. The steps are: (i) prepare; (ii) rationalize; (iii) implement; and (iv) consolidate. We also recommend the most appropriate techniques to be used in each step according to the scope (single or multiple specialties) of the STR study.

In step (i), the scope of the STR project is defined such that a single surgical specialty or a group of specialties will be selected for rationalization. The STR implementation team will be dimensioned according to the STR scope. For STR projects covering several specialties, we recommend one team to coordinate activities across specialties (general team) and several teams representing the surgical specialties (specialty teams). We recommend including surgical theater and sterilization unit managers in the general team, as well as a quality assurance staff member. Specialty teams usually include leaders of each surgical specialty, nurses, and nurse technicians, with meetings moderated by members of the general team. For STR projects covering a single specialty, the implementation team combines members of the general and specialty teams. Implementation teams will be responsible for collecting information available on the STs and defining project monitoring indicators. Regardless of project scope, we recommend the use of techniques that stimulate information sharing and support data collection; FG, BRT, and ITV are good choices since they do not require technical knowledge to be implemented.

Step (ii) is likely to be the more time-consuming in STR studies since it involves analyzing STs to promote reductions in the number of instruments they contain. Rationalization may take place at a tray level, by removing instruments that are historically not needed to perform procedures that use the ST, at a specialty level, identifying commonalities among procedures and assembling new trays that could simultaneously serve multiple procedures within a specialty, or at a system level, assembling new trays that could be used in procedures from different surgical specialties. Moving from tray level to system level increases the complexity of the analysis and the number of teams involved. For STR studies that focus on a single specialty, less complex techniques such as CL and L5S are recommended. In the case of multiple specialties, more complex techniques, such as HEU and CAN, may be required to cope with complexity. Main outputs in step (ii) are revised STs, the proposition of new STs that take advantage of commonalities between surgical procedures, and new assignments of STs to procedures.

In step (iii), the length of the validation period must be set. During that period, new STs will be sent to the ORs for validation. Outcomes are (a) further rationalization of trays, (b) request to include new instruments, or (c) the return of removed instruments to trays. We recommend using OBS, ITV, and CL to collect information in (a) to (c), and STD after validation is complete.

In step (iv), new documentation regarding STs will be formalized. The performance of the STR study regarding the project monitoring indicators established in step (i) will be determined and informed to rationalization teams and to the hospital’s upper management. We recommend using STD to produce the new ST documentation, CL and OBS to gather information on monitoring indicators, and SOP to consolidate an STR operational procedure for future studies.

## Conclusions

In this section, we address RQ4 through the proposition of a future research agenda. In our review, we identified some gaps in the literature related to STR. They are presented in Table 4 and in the next paragraphs as research questions associated with the STR steps proposed in section 5: (i) prepare; (ii) rationalize; (iii) implement; and (iv) consolidate.

**Table 4:** Future research questions associated with STR steps

Step	Research questions
Prepare	How to raise consensus among multidisciplinary teams to work on rationalization projects?
	How to encourage commitment in rationalization projects?
Rationalize	What technologies could be explored to manage instruments’ traceability?
	How can cross-sectional analysis of STs contribute to reducing instruments or STs?
Implement	What is the best solution to the instruments removed from trays?
	What are the intangible benefits of rationalization STs, and how to measure them?
	Which indicators can be used to measure the safety of surgical procedures following the rationalization of STs?
Consolidate	What strategies could be used to consolidate improvements achieved?
	What are relevant future goals to be set by organizations that already have been through an STR cycle?

Lack of consensus among surgical specialties’ leaders and low commitment to quality improvement programs that aim at the rationalization of STs are recurrent barriers reported when implementing step (*i*). When such programs are incorporated into the hospital’s routine involving all stakeholders, barriers are mitigated, and the process tends to be successful. Six Sigma projects represent a good opportunity for that, especially if having surgical specialties’ leaders as project champions.

Future research associated with step (ii) may be targeted at two promising directions: traceability of instruments and cross-sectional analysis of STs’ contents. Instrument traceability is important to ensure patient safety during surgery and to expedite the ST assembly process. Radio Frequency Identification (RFID) technology may be used for that. On the other hand, a cross-sectional analysis of STs’ contents allows taking into account commonalities in the use of instruments (within and across specialties) when rationalizing STs, further optimizing the process.

STR studies rarely report what was done with instruments removed from trays. However, the reuse of instruments has both environmental and economic impacts and should affect the way step (*iii*) is implemented. Some instruments are removed due to redundancy (and could be therefore reused in other STs), while others are removed for being rarely needed (and could be packed individually or in groups, being available at surgeons’ request). Step (*iii*) should be conceived to allow a clear distinction between these categories of instruments. Results from step (*iii*) rarely include indicators related to the safety of procedures using reduced STs and the intangible benefits of rationalization; e.g., reduced stress in the OR, simplified learning of procedures by new surgeons, improvements in the flow of surgical cases, especially when searching for instruments in high-stress situations. Those are promising research opportunities.

Once a rationalization cycle is complete, the implementation team should conceive strategies to consolidate improvements and set goals for future rationalization projects. Those issues are rarely addressed in reports on step (*iv*) and are open research opportunities.

## Data Availability

All data generated or analyzed during this study are included in this published article.

## Abbreviations

ABC: ABC costing
BRT: Brainstorming
CA: Chronoanalysis
CAN: Closest neighbor algorithm
CL: Checklist
CMS: Centers for Medicare & Medicaid Services
CP: Custom pack
CT: Cost tray
DT: Decision tree
EA: Expert analysis
FG: Focus groups
GDP: Gross domestic product
HEU: Heuristic
ITV: Interview
KAI: Kaizen
KDD: Key driver diagram
L5S: Lean 5S
LP: Lean practices
LP: Linear programming
MILP: Mixed integer linear programming
MP: Mathematical programming
NG: Nominal Group
NIP: Nonlinear integer programming
OBS: Observation
OCDE: Organization for Economic Cooperation and Development
OR: Operating room
OSW: Ohno’s seven waste
PC: Preference cards
PDSA: Plan, Do, Study, Act
PF: Process flowcharts,
PLM: Modified integer linear programming
PRISMA: Preferred Reporting Items for Systematic Reviews and Meta-Analysis
PSI: Patient-specific instrumentation
RCA: Root cause analysis
RQ: research question
SG: Surgical Manual
SIT: Shadowed instrument tray
SOP: Standard operational procedure
ST: Surgical tray
STD: Standardization
STR: Surgical tray Rationalization
SXS: Six Sigma
TDI: Template-directed instrumentation
VSM: Value stream mapping
